# Dr. Quain's Elements of Anatomy

**Published:** 1835-01-01

**Authors:** 


### The Elements of Anatomy. By *Jones Quain* M.D. Professor of Anatomy and Physiology in the University of London. Third Edition, revised and enlarged.

The comparative rapidity with which this work has passed through two editions, and attained a third, is primâ facie evidence of its deserts. The popularity of Mr. Quain as a teacher of anatomy, and the enormous class which he is said to instruct, contribute, beyond doubt, to promote the sale of the system before us.

Perhaps we may be permitted to avail ourselves of the present opportunity of offering a few remarks on the subject of anatomy. It is impossible for any who are conversant with the method of teaching now practised in the *best* metropolitan schools, to avoid perceiving the great alteration which that method has experienced in the last few years. Mr. Abernethy, we believe, was the first to adopt a slip-slop mode of lecturing, in which minute and rigid demonstration was sacrificed, and a very superficial description of anatomy was patched and garnished with trimmings of physiology, pathology, and therapeutics. The lecturers of the Abernethian school resembled very closely that liberal cook, who offered for threepence a dinner consisting of "roast and boiled." He kept his word; but, when the covers were removed, potatoes were the only dish.

The abandonment of minute anatomy, and the association of pathology and surgery along with it, offered a short cut and an easy path to the deluded student. The lecture-room and manual were occupied alike with this very promising and very profitless species of instruction. Much was said, but little taught; and if the student had not the fortune to be made a jack of all trades, he certainly was not a master of anatomy.

The publication of the work of M. Cloquet, and its speedy appearance in an English form, effected a rapid, if not total, overthrow of the system of lecturing to which we have adverted. Exactness of description and precision of information took the place of the vague generalities of that system, and the retail rubbish of pathology and surgery were excluded from the anatomical theatre.

We do not affirm that the superficial lecturers are entirely defunct, or that some do not still neglect their proper business—the teaching of anatomy, for the purpose of foisting their cheap and nasty specimens of pathology and surgery upon their pupils. Yet such lecturers are not popular; they are shunned as idle praters or absolute impostors, and the race of *fainéants* must quickly disappear. Peace to their manes! They belong as certainly to a former age, as if they had been born two centuries ago.

The work of Mr. Quain is not absolutely free from the contagion of the period now happily gone by. There is in it a disposition to mix up other things with the actual subject, to fit up the dissecting-case with the matériel of surgery. The forms of hernia, the taxis, the operations are introduced—the history of lithotomy is given—the modes of securing the arteries are detailed. We hold that it is better for each to confine himself to his own occupation—for the lecturer on anatomy to stick to that—and the lecturer on surgery to teach surgery only. Each were wise to arrange and display his appropriate wares, rather than to filch some trivial and some shewy article from his neighbour's stock.

Ne sutor ultrà crepidam.

As an instance of the mischief resulting from this dabbling in other persons' ponds, we would cite Mr. Quain's hasty notice of the recto-vesical operation, for extracting a stone from the bladder.

"The *recto-vesical* operation consists in laying open the bladder in the middle line by cutting from below upwards, beginning in the rectum, and so laying the two cavities into one. I mention it merely because it has been practised by M. Sanson in Paris, and M. Vacca Berlingieri in Pisa. It is not sanctioned by any surgeon in this country, nor is it likely to meet with much approval, as it contravenes one of the first principles of surgery, which prescribes that we are not to wound any important parts which can be avoided." 461.

It is not correct that the recto-vesical operation is not sanctioned by any surgeon in this country. Sir Benjamin Brodie performed it two or three years ago. And it would not be difficult to shew that circumstances occasionally justify, or even require its selection. We might cite other instances of the dubious character of the surgical observations in the present work. It is enough to shew, that such "double skimm'd skie blue" as an anatomical lecturer can afford, we mean in point of space, is neither useful nor ornamental. We would strongly advise Mr. Quain to strike out the surgical patches from the volume, when it reaches, as it soon will do, the fourth edition. It is sufficient praise to write a good treatise on anatomy. The credit is rather diminished than increased, by the negative addition of some very common-place surgical remarks.*

It may seem that we have dealt over much in censure. If so, it has been kindly meant, with the view and in the hope, that Mr. Quain would purge his excellent work of almost the only materia morbi about it. But we cannot part from the author or our readers without a more cordial introduction, and a recommendation that each should grow thoroughly acquainted with the other. As an instance of the instruction derivable from a close acquaintance with the former, we may cite a passage selected without care, and calculated to display the general features of the volume. It is the narrative of the course and description of the sources of the internal jugular vein.

"Internal Jugular Vein.

*Vena jugularis interna.*—The blood from the brain and cranial cavity is received by the internal jugular veins, which are continuous at their upper extremities with the lateral sinuses; whilst inferiorly, they terminate in the brachio-cephalic veins. The junction of the vein with the sinus is at the broad part of the foramen lacerum *(fossa jugularis)*, beneath which it is supported by the rectus laterales muscle, and lies close at the outer side of the internal carotid artery, as far as the cornu of the os hyoides. Now, as the part of the vessel which extends from the skull to this point corresponds with the internal carotid artery, and receives its residual blood, it may with much propriety (and also with advantage, as tending to render the nomenclature uniform) be called *vena carotis interna.* Some confusion would arise from calling it 'cephalica,' as the external superficial vein of the arm has, without any proper reason, received that name. Again, the short vessel which extends downwards from the junction of the facial and temporal veins and joins the preceding, may, for the like reason, be called *vena carotis externa;* and finally, the trunk which results from the conflux of these vessels, and which extends thence down to the inner end of the clavicle, should be called *vena carotis communis.* At present, the entire length of the vessel included between the point just named and the base of the skull, is known as the internal 'jugular' vein, as if it belonged to the throat, and had no correspondence in its divisions and distribution with the artery which it accompanies. However, we must defer to usage, and continue for the present so to name it.

The internal jugular vein having passed down to a level with the os hyoides, receives the common trunk formed by the facial and temporal veins, and then

---

* Mr. Quain may fall back on the example of Mr. Harrison. The worst part of that gentleman's excellent work upon the arteries is its surgical cement.

becomes considerably enlarged. It descends parallel with the common carotid artery, lying at its outside enclosed in the same sheath, together with the vagus nerve, and at the root of the neck joins nearly at a right angle with the subclavian vein, and so forms the innominata, or brachio-cephalic. Previously to its junction with the facial vein, the internal jugular receives branches from the tongue, pharynx, and occiput :—*Vena lingualis* commences at the side and upper surface of the tongue, passes backwards, receiving branches from the sublingual gland; occasionally the ranine vein joins it, sometimes also the pharyngeal. In either case it passes backwards between the mylo-hyoideus and hyo-glossus muscles, to open into the jugular vein. *Vena pharyngea* commences at the back and sides of the pharynx, and terminates in the lingual, or separately in the jugular vein. *Vena occipitalis* corresponds in its course, and in the distribution of its branches, with the artery of the same name. It communicates with a plexus of veins on the outside of the occiput, and terminates occasionally in the external jugular vein, but more frequently in the internal.

*Vena laryngea* is a small vessel whose branches come out of the larynx through the thyro-hyoid membrane ; they unite and form one vein, which opens into the internal jugular (its anterior or facial division) : sometimes it terminates in the superior thyroid vein.

*Vena thyroidea superior* arises by ramusculi, which ramify in the thyroid gland, in company with the branches of the superior thyroid artery. These unite and form a single vessel, which runs transversely outwards, and open into the jugular vein (its common trunk.) Lower down we find another branch coming from the source *(vena thyroidea media.)*" 578.

